# Phelgmasia Cerulea Dolens Diagnosed by Point-of-Care Ultrasound

**DOI:** 10.5811/cpcem.2016.12.32716

**Published:** 2017-03-13

**Authors:** Michele Schroeder, Amanda Shorette, Sukhdeep Singh, Gavin Budhram

**Affiliations:** University of Massachusetts Medical School-Baystate Health Springfield Campus, Baystate Medical Center, Department of Emergency Medicine, Springfield, Massachusetts

## Abstract

Phlegmasia cerulea dolens (PCD) is a rare entity that is associated with significant morbidity and mortality, including limb ischemia and pulmonary embolism. Point-of-care ultrasound (POCUS) can expedite the diagnosis, leading to earlier life- and limb-saving treatment. Although primarily used for assessing for the presence of deep venous thrombosis, in the appropriate clinical setting POCUS can also be used to diagnosis PCD as well as to distinguish between venous and arterial occlusion, which can lead to a difference in management. We present a case of phlegmasia cerulea dolens after mild trauma in a patient with an underlying hypercoagulability disorder diagnosed by an emergency physician using POCUS, which expedited treatment with catheter-directed thrombolytic therapy.

## INTRODUCTION

Phlegmasia cerulea dolens (PCD), or massive proximal venous thrombosis of the lower extremity, is a rare entity that is associated with significant morbidity and mortality, necessitating rapid diagnosis and treatment. Point-of-care ultrasound (POCUS) can expedite the diagnosis, leading to earlier life- and limb-saving treatment. PCD is most often associated with hypercoagulable states, but can be precipitated by trauma. We present a case of phlegmasia cerulea dolens after mild trauma in a patient with an underlying hypercoagulability disorder diagnosed by an emergency physician using POCUS, which expedited treatment with catheter-directed thrombolytic therapy.

## CASE REPORT

A 21-year-old female college student presented to the emergency department (ED) with severe pain in her left hip, thigh, and calf. The pain started suddenly when she was playing basketball a few hours earlier. She twisted her torso to shoot the ball and heard a “pop” in her left hip with immediate onset of pain, and she subsequently fell to her knees. She was initially able to ambulate but had worsening pain and rapid progression of swelling and mottling in the left leg from her hip to her ankle. She denied any pain or swelling in that extremity prior to the injury during the basketball game. With the exception of oral contraceptive use, she had no significant medical or family history.

On examination her left extremity was mottled, dusky, and cool to the touch from the hip to the toes and she had decreased dorsalis pedis and posterior tibial pulses when compared with the right extremity. The leg was markedly tender to palpation and sensation to light touch was diminished. Passive range of motion of the hip, knee, and ankle elicited severe pain.

**Image 1:**
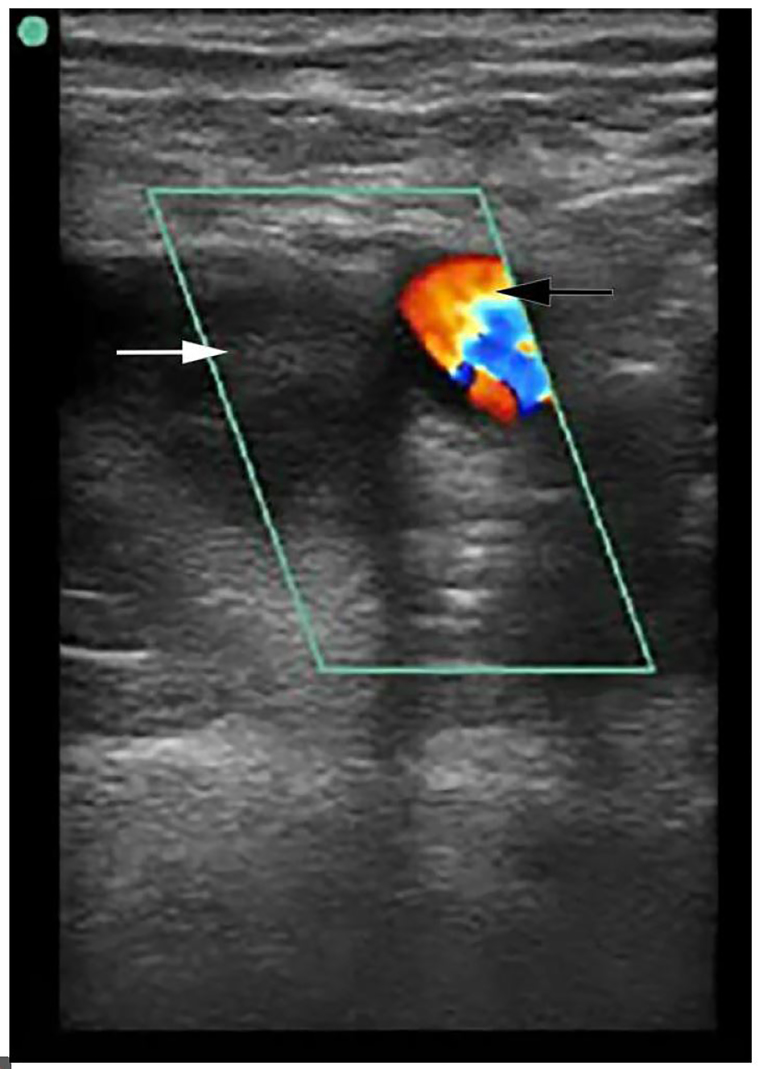
Point-of-care ultrasound demonstrating complete thrombotic occlusion of the femoral vein. In this still image, application of color Doppler shows normal flow through the femoral artery (black arrow) but absence of flow in the femoral vein. Echogenic material is visualized within the femoral vein (white arrow).

**Image 2A and 2B:**
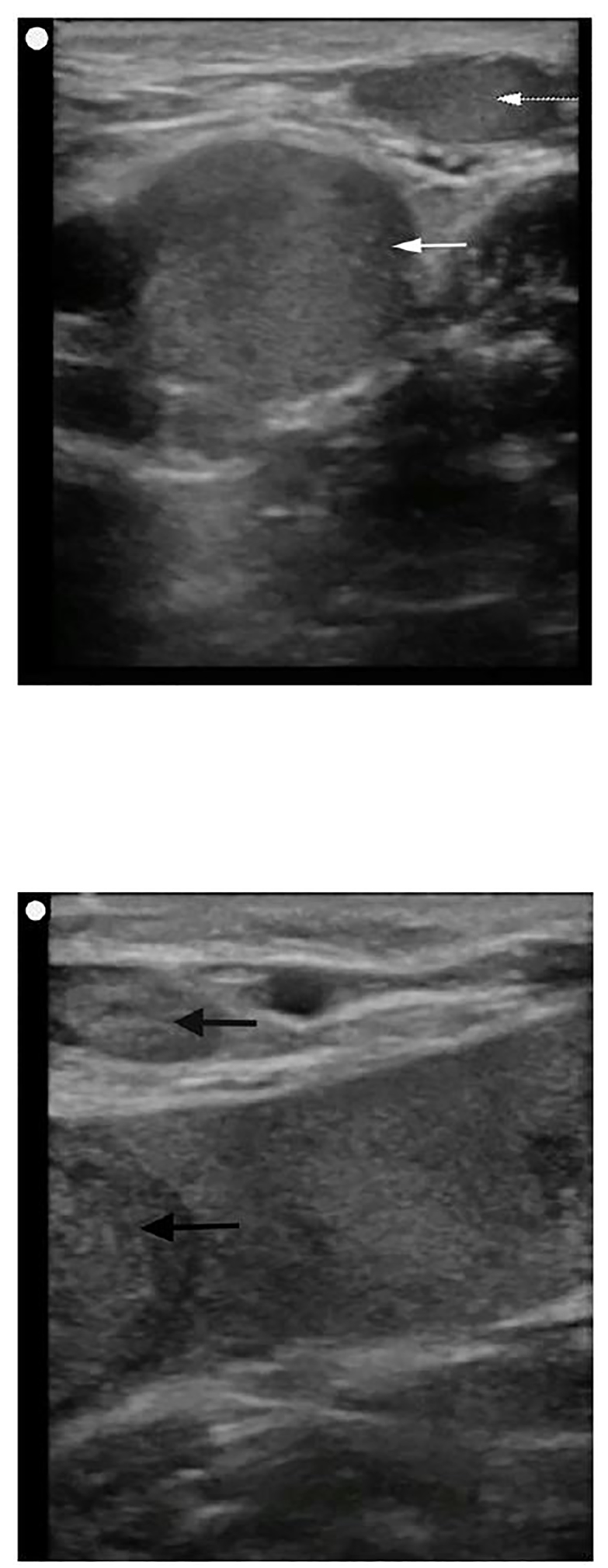
Point of care ultrasound demonstrating extensive venous thrombosis of the left lower extremity. In image 2A, echogenic material is seen within the left common femoral vein (white arrow) and the greater saphenous vein (dashed white arrow). In image 2B, more echogenic material was visualized distally within the popliteal veins (black arrows).

**Image 3:**
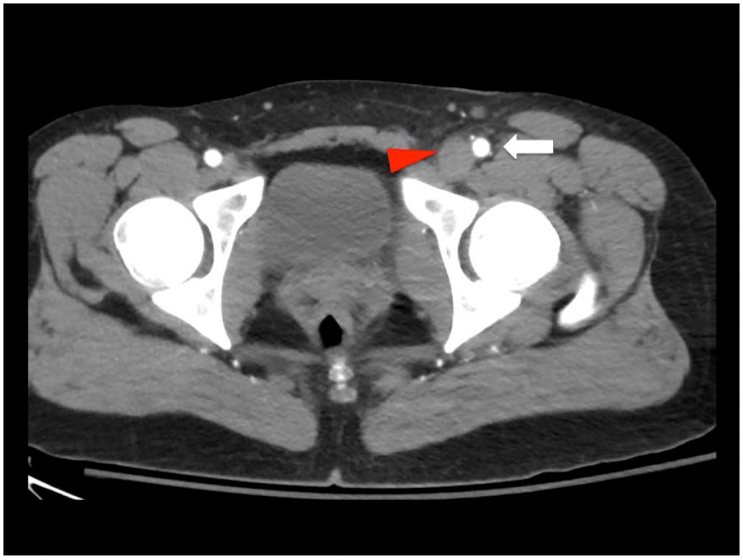
Axial view of computed tomography angiogram at the level of the common femoral artery and vein. The femoral artery is patient (white arrow) while the common femoral vein is distended and filled with clot (red arrow).

POCUS was performed at the bedside to evaluate for arterial blood flow and deep venous thrombosis. This demonstrated normal color flow in the femoral and popliteal arteries (Image 1), but echogenic material was noted within the left common femoral vein extending distally to the popliteal vein and the veins were non-compressible (Images 2A and 2B). Vascular surgery was immediately consulted, and while awaiting their arrival to the ED the patient was sent for an emergent computed tomography (CT) angiogram, which confirmed arterial patency and extensive deep venous thrombosis in the left popliteal, femoral, and iliac veins (Image 3). A heparin bolus was given and the patient was then taken to interventional radiology where she underwent partial thrombectomy, catheter-directed tissue plasminogen activator (tPA) treatment, and placement of an inferior vena cava (IVC) filter. By the next morning her leg was noted to be well-perfused, non-tender, and without swelling, and her pain had resolved. A workup for hypercoagulable states revealed heterozygosity for the Factor V Leiden mutation. She was discharged one week later on warfarin after bridging from enoxaparin.

## DISCUSSION

PCD is a rare form of deep venous thrombosis in which acute massive proximal venous thrombosis results in obstruction of the venous drainage of an extremity. Symptoms include sudden severe pain, swelling, edema, and cyanosis, leading to venous gangrene and compartment syndrome.^1^, ^2^ Prompt diagnosis and treatment is necessary to return circulation and prevent circulatory collapse, and a delay in treatment may result in loss of the patient’s limb or even death.^3^,^4^ The condition can have either a gradual or a fulminant course.^5^

Most cases of PCD are associated with an underlying hypercoagulable disorder, most often malignancy.^6^ There are case reports, however, of fulminant PCD resulting from trauma, including one patient with a hip dislocation.^7^, ^8^ The traumatic injury is unclear in the current case as the ED imaging was negative for any fracture or dislocation, although hip subluxation/dislocation with spontaneous reduction has been described in adolescents.^9^ This patient reported a “popping” sensation suggestive of this mechanism of injury and she additionally reported a fall from standing immediately after the onset of pain. This mild trauma in conjunction with an underlying hypercoagulability disorder is the likely etiology of her massive lower extremity venous thrombosis.

Angiography at this stage will typically demonstrate patent arterial flow as clinical mottling occurs due to peripheral vasospasm and severe edema from thrombosis. PCD can mimic acute arterial occlusion, but rather than a lack of blood inflow, venous gangrene results from stasis due to lack of outflow. Both conditions are initially treated with heparin, but since massive pulmonary embolism is an immediate threat with PCD, procedural interventions such as thrombectomy, catheter-directed tPA and IVC filter placement may be warranted even if perfusion improves with heparin.^10^, ^11^

The diagnosis of PCD employs a combination of clinical and radiologic findings. Manifestations of phlegmasia alba dolens (the clinical precursor to PCD) include edema, pain and blanching. As the disease progresses, cyanosis develops, and can lead to bleb and bullae formation and venous gangrene.^12^ Ultrasound is commonly used to identify venous thrombosis, and a POCUS performed by an emergency physician can quickly establish this time-sensitive diagnosis. Previous studies have demonstrated that POCUS performed by emergency physicians is sensitive (89–100%) and specific (75–99%) when compared to radiology department ultrasound for the evaluation of deep venous thrombosis.^13^–^17^ The clinician should be aware, however, that thrombosis of the iliac veins may not be seen by ultrasound. CT venography is commonly used to diagnosis proximal thrombosis but is not always readily available at some institutions.

In this patient, massive lower extremity venous thrombosis was easily visualized by the emergency physician using POCUS. Further support for the diagnosis in this patient (and against arterial insufficiency) was the presence of Doppler flow in the arterial system. While ultrasound alone may not be able to distinguish DVT from PCD, the finding of non-compressible proximal veins in combination with a mottled, cool, and painful extremity should be sufficient to raise the suspicion of PCD.

## CONCLUSION

PCD is a rare disease with significant morbidity and mortality. Early diagnosis and treatment is necessary to prevent severe complications such as limb ischemia or massive pulmonary embolism. Point of care ultrasound can facilitate earlier diagnosis of significant vascular pathology, including phlegmasia cerulea dolens.
